# Public-private partnership in the Portuguese health sector

**DOI:** 10.1016/j.heliyon.2023.e19122

**Published:** 2023-08-14

**Authors:** Nuno José Pinho Rodrigues, João M.S. Carvalho

**Affiliations:** a Instituto Politécnico de Gestão e Tecnologia, V. N. de Gaia, Portugal; b REMIT-Universidade Portucalense, R. António Bernardino de Almeida, 541, 4200-072, Porto, Portugal; c REMIT-Universidade Portucalense, Porto, Portugal; d R. António Bernardino de Almeida 541, 4200-072, Porto, Portugal

**Keywords:** Public management hospitals, Public-private partnerships, Health service quality, Value-for-money, Public sector comparator

## Abstract

Since 2001, in Portugal, constant reforms in hospital management have accompanied the transformations in the management models applied to public administration, intending to ensure a higher quality of services and, simultaneously, a more significant economic efficiency. This study aims to analyse, for the period between 2012 and 2021, the economic and financial results (value-for-money) of the PPP model, compared with the public management hospitals (PMH). It used a mixed research approach based on multiple case studies and archival research. As the main results, it was found that: i) the PPP model, applied to the health sector, appears to be advantageous, not only regarding the economic and financial results but also concerning the quality of service provision; and ii) despite the value-for-money generated by the PPP model, the lower operating costs and the superior performance in comparison with PMH, the government has permanently opted to revert from a PPP model to a PMH model. This study concluded that the hospital management model is instead seen as an instrumentalised political instrument than a management tool that could generate savings for the taxpayers. Several practical implications are presented.

## 1 Introduction

To employ their influence on the economy and society, governments have many instruments at their disposal, ranging from simple rules or procedures to complex tax and benefit systems, which are often not fully understood [1]. The relevant factors for the definition and choice of governance instruments are likely to vary with not only the background and functions of the decision-makers but, even more importantly, with their cognitive orientations and specific characteristics of their organizational context [1].

Governments operate as economic agents, responsible for providing and managing essential goods and services, such as education, security, and health services. However, due to ongoing inefficient management of public resources and/or budgetary constraints, there are historical difficulties in providing these goods and services [[2], [3], [4]].

Investments in public infrastructure for social services may involve some form of partnership regulated by a contractual agreement with private partners. For instance, concession contracts, availability-based contracts, or traditional public contracts are the first two usually considered an instrument called Public-Private Partnership (PPP) [5]. Carvalho [6] argued that public services are protected from competitive pressure. However, many public sector activities (including health) have been handed over worldwide to the private and third sectors, allowing competition to occur. In this context, PPPs have been used to construct and operate infrastructure and social facilities [[7], [8], [9]]. The PPP model involves the award, purchase, or subcontracting of public services to private partners through different contractual arrangements [10]. Unlike the privatisation model, which involves the sale of public goods/assets to private owners, the PPP model currently includes different contracts/models with the private sector. This type of contract ranges from short-term contracts involving only part of the service provided (e.g., contracting, operation, and maintenance) to more ambitious, long-term agreements that apply to an entire service (e.g., leasing or concession) [10,11].

Despite the proliferation of the PPP model, there is no consensus among academics regarding its application [12]. Among several definitions in the literature [e.g., [[13], [14], [15]]], OECD [16, 17] defined PPP as an agreement between the Public Sector and one or more private entities. The latter provides a service that meets the requirements defined by the Government and, at the same time, generates profit for shareholders, being these two requirements depend on the risks allocated to each party. There are several typologies applicable to the PPP model. However, based on the contract, the Design-Build-Finance-Operate (DBFO) model has been a general approach [17]. In the DBFO model, the State defines the services it wants the private sector to provide, leaving the private sector to finance and build the actual asset. In addition to construction, the private partner manages the asset and provides its services [18]. The DBFO model was chosen by the government to be implemented in the PPP hospitals, including the construction, financing, and maintenance of the building, the responsibility of operating the clinical services, and the transfer of property to the State at the end of the contract.

### 1.1 Theoretical framework

The literature argues that traditional public procurement of infrastructure provision/operation has resulted in severe cost overruns and delays [19]. These arguments favour the use of the PPP model instead of conventional public procurement models because the private involvement in the design, financing, construction, and operation phases could reduce the costs of the entire project lifecycle and/or provide higher quality for the same resources. These results mean a higher Value for Money (*VfM*) [20,21]. Thus, based on the need for a correct assessment of *VfM*, the literature argued that a successful PPP implies intended objectives achievement, such as providing sufficient quantity and quality public goods and improving efficiency and *VfM* [22]. Despite this idea, previous studies on the costs, quality, and *VfM* in applying PPP models for infrastructure development suggest mixed results [e.g., [23]]. Some analyses revealed lower costs and/or higher *VfM* through applying the PPP model [e.g., [24]], while others reached the opposite conclusion [e.g., [25]]. Many researchers have tried to point out ways to improve the functioning and operation of projects conducted under this model, identifying critical aspects of this project [e.g., [[26], [27], [28]]]. For example, Edkins and Smyth [29] pointed out that the contract terms may be a key factor, as they generally significantly impact relationship performance. Akintoye et al. [7] found that the high cost of the procurement process is a crucial factor loading on the PPP project and leading to a reduction in the private sector's willingness to participate in it. However, how can we measure *VfM*? According to Her Majesty's Treasury [30], *VfM* is the optimal combination of the life cycle costs of a given project and the quality of the good or service that aims to satisfy user requirements, including risk assessment and time to completion [21]. Thus, we can conclude that when a PPP is a way to finance and operate public infrastructure and services, then the *VfM* should be calculated based on the Public Sector Comparator (PSC). The PSC can be defined as the best project that can be carried out and financed directly by the State, through public procurement, to perform, with all the specified requirements, the provision of the service and achieve the same objectives as the project developed under the PPP regime [3,31]. Additionally, for Cruz and Sarmento [32], PSC is an economic model of project valuation, i.e., all life cycle cash flows are discounted to an NPV, and their sum represents the life cycle NPV of a given infrastructure. Finally, Sarmento [33] argued that the PSC is the financial difference between the two award options for the same project, becoming a negotiation tool for the public sector, which may lead to the best possible agreement with the private sector.

In the last decade, there has been a proliferation of different PPP modalities to provide public services such as health in developed and developing countries [34]. Ramanadhan et al. [35] investigate how telehealth can enhance access to high-quality healthcare for rural populations in India, highlighting the prominence of taking a systems perspective and engaging inter-sectoral partners through the alignment of values and goals. Additionally, the creation of a synergistic, health-promoting ecosystem offers the potential to support telehealth services in the long-term. Also, Ganapathy et al. [36] argued that healthcare is provided in developing countries, with a lack of infrastructure and personnel, leading the governments to realize that digital health through PPP could mitigate the issue. The authors concluded that given a committed collaborative team digital health in a PPP mode in a developing country is highly applicable.

The private sector's provision of infrastructure or public services carries out a significant risk and management responsibility [20]. In this context, PPP models are increasingly seen as a tool to improve the performance of health systems worldwide by bringing together the best features of the public and private sectors to improve efficiency, quality, and innovation [7,13]. Considering the need to renew infrastructure and increase the quality of services, the PPP model has been promoted as an effective strategy to meet patients' demands in terms of quality of service [37]. The participation of private capital in the healthcare market through the PPP model could be a viable and sustainable approach to reducing the gap between the demand and supply of adequate healthcare services [38]. More, taking as the principle that the goal of Hospitals is to promote the health of the population so that they can actively participate in the economic and social activities of the country [39]. Thus, this research is justified by the existence of an open debate on the PPP model advantages and the consequent estimation of the benefits obtained by their use [e.g., 8, 13, 40], particularly in the health area. In addition, the PPP management model is a motivator of controversy among academics [e.g., 8, [[40], [41]]], which translates, in many situations, into a dissonance between purely economic-financial analysis to the model and the perception of quality of the service provided. This situation is critical when the government needs to decide on this management model regarding either its adjudication or consequent renewal. There is also a significant gap in knowledge due to the scarcity of research analysing the impact of the PPP model concerning service quality. As Hellowell [42] argued, there is little guidance for decision-makers on the circumstances in which the PPP model is likely to produce good results. Caballer-Tarazona and Vivas-Consuelo [20] argued that even though the PPP model generates great interest in the academic world, the existing literature is still fragmented and focused on the management cost aspect. As such, especially in the health sector, a holistic analysis of hospital performance is required, and not only a plain cost analysis. A key factor when choosing the contracting model for the construction and operation of a given infrastructure (e.g., a Hospital) is the assessment of the quality/price ratio or *VfM*, representing an expression of the savings resulting from the efficiency and effectiveness of the service received by a public entity [8]. At the same time, the PPP is expected to fulfil its intended social and environmental objectives, such as equity for underdeveloped areas, affordability for people with low incomes, stakeholder satisfaction, environmental protection, and resource conservation [22].

For Tuohy [43], the PPP model represents a repositioning of health systems by governments, uniting the logic of effectiveness and efficiency, focusing on three-dimensional objectives: (i) to allow public entities to access private capital and better management; (ii) to allow the public sector to benefit from the experience of the private sector and for the State to access technologies in a less costly manner and, above all, to redistribute risks, the security of service provision and the extension of health infrastructures and services; and (iii) to use, in an intelligent manner, the flexibility and exactitude of the private sector to counterbalance the slowness of public sector regulations and procedures, thus allowing a better adjustment of the State's means to its objectives.

Proponents of the PPP model indicate the superiority of PPPs over public healthcare providers, arguing about the better productivity of the model. However, international evidence is inconclusive, particularly regarding the perceived quality of care [e.g., [44]]. Notably, despite several evaluations of both private and public entities in Portugal, no clear conclusion has yet been reached on the PPP model in health. However, there are common points in several published reports: one is that PPPs generate savings for the State; the other is that there is a lack of indicators for comparison with other public hospital management models.

According to the Basic Law on Health (Law no. 95/2019), which is currently in force in Portugal, Public Hospitals can be managed either through public management indicated by the State or through a PPP model [45]. Thus, this study aims to answer the following questions.

1. In the period between 2012 and 2021, what are the *VfM* of the hospitals under a PPP model?
2. Is the performance (health care) registered in the period 2012–2021 in the PPP hospitals inferior or superior to the performance of the public management hospitals (PMH)?

This research was done based on a multiple case study, collecting data using the archival research method.

The answers to the above questions are aimed to contribute to the PPP theoretical rhetoric clarification, especially regarding the actual results achieved throughout the entire project life cycle in terms of *VfM* and performance.

## 2 Methods

To answer the first research question, one used a document analysis of all official state reports, where the operational and financial results of the four PPP Hospitals are reported, as well as the *ex-ante* analyses carried out by the public partner, as provided in the article 6.1 of the Decree-Law No. 111/2012. This Decree-Law was the first cross-cutting legislative initiative aimed explicitly at PPP, seeking to enhance the public sector's use of the management capacity of the private sector, improve the quality of the provided public services, and generate savings in the use of public resources. The *ex-ante* analysis was conducted to assess *VfM* using a complete cost-benefit analysis and the Public Sector Comparator (PSC), as proposed by Sarmento [33] and Garvin [46]. We identified several sources of information, public and private, that will allow us to assess the economic and financial results of PPP projects, namely the reports periodically produced by the Technical Project Monitoring Unit (UTAP) and the Technical Budget Support Unit (UTAO). In addition, the advice issued by the Audit Office Court (AOC), and the financial studies produced over the years by several independent private entities, were analysed.

The second research question was approached by a qualitative and quantitative analysis of PPP Hospitals' performance indicators (e.g., costs, production capacity, quality, etc.) compared to PMH, building on the methodology applied by Caballer-Tarazona and Vivas-Consuelo [20] in Spain.

**Fig. 1:**
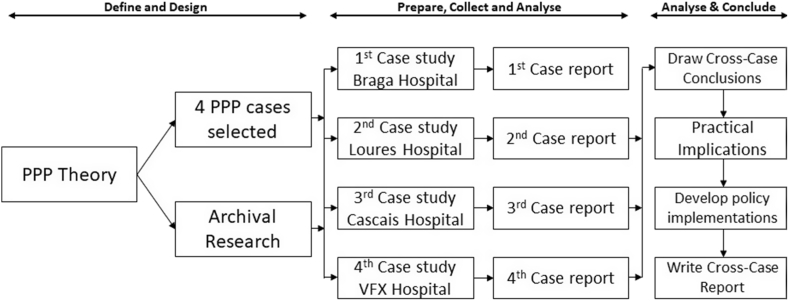
Multiple case study design.

The research methods were applied on a multiple case study of Portuguese hospitals (Fig. 1). It is the most appropriate approach to the fact that there are (or have been) only four hospitals under the PPP model in Portugal [47]. Case studies are widely used in organizational studies and social sciences [48]. They should have clear designs, produced at a time before any data is collected. These designs should cover the main research questions, the unit of analysis, links between data, and data interpretation procedures [49]. Fig. 1 shows the life cycle of this multiple case study research.

This study chose the archival research method, which can be defined as secondary data that are collected and stored before research begins, intended for later use. Archival data can include statistical information, court files, institute records, credit histories, and educational records. Business archival data may consist of annual reports, analysis files, and responses to surveys conducted at different times [50].

For data collection to be reusable, it must be ‘processed’, involving a series of activities: verification, digitisation, optical character recognition, conversion, anonymisation, organisation, and metadata creation [48]. All these activities were performed, being the data set verified regarding the sources and scope of the documents and converted into a data list allowing correlations between all the information regarding the health PPP model management in Portugal. All collected data have public access; thus, anonymisation was unnecessary.

### 2.1 Sampling

Eight cases are included in this multiple case study, including the four PPP hospitals in Portugal, and the four PMH chose to make comparisons about performance indicators. Three of the PPP hospitals are installed in neighbouring municipalities of Lisbon: Cascais (5th large municipality), Loures (6th), and Vila Franca de Xira (17th). The fourth one is Braga, in the North of the country, and is the seventh large municipality of Portugal.

Comparing PMH and PPP hospitals is a complex exercise, mainly due to their heterogeneity. Clause no. 43 of the PPP Management Contracts considers the comparison of PPP Hospitals with other National Health Service (NHS) Hospitals that integrate the same Group (clusters). These hierarchical clusters are defined by the Central Administration of the Health System [51] according to their characteristics, namely based on a principal component analysis of their standardised cost variables. This hierarchical clustering model doesn't have a group A. The others are categorised as follows: B, C, D, E and F. Only groups B, C and D were relevant for this study, as they contain the four PPP hospitals. It was chosen to compare with Vila Franca de Xira hospital in group B the Figueira da Foz hospital; in the group C (Cascais and Loures hospitals) the Setúbal and Leiria hospitals; and in the group D (Braga hospital) the Évora hospital. Thus, PPP hospitals were compared with PMH with similar characteristics, meaning hospitals belonging to the same group [[52], [53], [54], [55], [56]].

### 2.2 Variable measures

In this study, hospital management models were assessed by *VfM* and Service Performance. The first variable allowed us to evaluate the results from the PPP model application in Portugal. The second variable gave us an insight into the performance achieved by each PPP hospital and the possibility of comparing the results with the PHM Hospitals. Based on these two variables, the State decides about the reversal or the renewal of the PPP contracts.

*VfM* is a concept that refers to the optimal use of resources, and it was calculated by combining three criteria: (i) Economy – cost minimization; (ii) Efficiency – the relationship between results and resources; and (iii) Effectiveness – the relationship between the intended results and the results obtained. As suggested by Cruz and Sarmento [32], one can determine the function *VfM* (Equation (1)), which is directly proportional to utility (μ) and inversely proportional to cost (c).

1 VfM=f(Δμ/Δc)orVfM∼f(μ,c−1)

The indicators that measure the variable ‘Service Performance’ are described in Table 1 in three dimensions: Health Care Performance, Costs Performance, and Access Performance.

**Table 1:** Measurement indicators.

Dimension	Indicator	
Health Care Performance	A	% Surgeries performed in the outpatient department in the total programmed surgeries for outpatient procedures
	B	% Readmissions within 30 Days
	C	% Hospitalisations with a delay of more than 30 days
	D	% Births by caesarean section
	E	% Hip fractures with surgery performed within the first 48 Hours
	F	% Caesarean section deliveries in single, cephalic, full-term pregnancies
	G	% First caesarean sections in single, cephalic, full-term pregnancies
	H	% Vaginal deliveries after caesarean section in single, cephalic, full-term pregnancies
Costs Performance	I	Operating costs per standard patient
Access Performance	J	% 1st consultations carried out in time
	K	% Surgical enrolment list, within the guaranteed maximum response times

The Health Care Performance was measured by considering all the eight indicators defined by the Central Administration of Health Systems [51]. The Cost Performance dimension has four indicators defined by CAHS [51]. However, there was only data available for one indicator given the limitation stated by the General Inspection of Health Activities [57, p.12]: “GIHA concluded that CAHS does not have complete information regarding the dimension of the cost for three PPP hospitals (Cascais, Loures, and Vila Franca de Xira)". The third dimension – Access Performance – has two indicators, defined by CAHS [51], considered fundamental to enhance the comparative study between the PMH and PPP Hospitals.

### 2.3 Data processing

The analysis of the variable ‘Service Performance’ was conducted using the database integrated into National Health Evaluation System (NHES) over the years 2012–2021 (https://benchmarking-acss.min-saude.pt/BH_Enquadramento/DimensaoBenchIndicadores). The analysis of the variable *VfM* was done by resourcing documentary information concerning investment, cost, production, and production capacity of the four PPP hospitals for that period.

Over the past two decades, it was witnessed large-scale investment in making archives and repositories available that capture an avalanche of new data. In addition, one can see a tremendous innovation in comprehensive methods for reusing qualitative data [e.g., [[57], [58], [59], [60]]]. This study's data were collected from documentary and archival records as sources of evidence [48], including secondary data (collected by other authors) and tertiary data (already analysed by other authors) [61,62].

To be successful in this research methodology, one must elaborate on three crucial documents [48], which will allow both the discovery of relevant data resources and informed reuse. The first is a systematic ‘Catalogue Record’ that provides a detailed overview of the study, the size and content of the dataset, its availability and any terms and conditions of access. The second is a ‘User Guide’, which brings together key documentation from the research, containing information on how to use the data, how the data were collected, and the original topic guides and publications. Finally, a ‘Dataset Listing’ with 60 documents, detailing key features of the data, was constructed to help readers identify particular types of data (Table 2). In many ways, these unique characteristics are analogous to 'variables' in quantitative data sets [48].

**Table 2:** Dataset listing.

Organizations	Type of document
Audit Office Court	Guidelines and Procedures 2008. Audit Report 2009, 2013, 2014, 2015, 2016, 2019, and 2021.
European Audit Office Court	Audit 2018.
Strategic Analysis Team	Final report 2008.
General Inspection of Health Activities	Benchmarking report 2013.
Health Regulatory Authority	Study 2016. Report 2020.
IASIST-IQVIA Economic and Clinical Information	Report 2016.
Portuguese Observatory on Health Systems	Report 2018.
General Inspection of Health Activities	Control and Monitoring of Hospital Management Contracts PPP 2018.
National Health System	Report 2018.
Technical Budgetary Support Unit	Technical Information 2015. Budget execution report 2015a, 2015b, 2016a, 2016b, 2017a, 2017b, 2018, 2019, and 2020.
Project Monitoring Technical Unit	Report 2016a, 2016b, 2016c, 2017a, 2017b,2017c, 2017d, 2017e, 2018a, 2018b, 2018c, 2018d, 2019a, 2019b, 2019c, 2019d, and 2019e. Management contract 2008, 2009a, 2009b, and 2010.
The Organisation for Economic Co-operation and Development	“Better Policies” Series 2013.
European PPP Expertise Centre	Official document 2011 and 2012.
United Nations	Official document 2008.
European Union	Official document 2013.
Catholic Lisbon University	Official document 2016.
PricewaterhouseCoopers International Limited	Official document 2013.
Health, Nutrition, and Population – World Bank	Official document 2006.
Republic Diary	Decree-Law n° 86/2003; Decree-Law n° 111/2012; Decree-Law n° 75/2019. Decree-Law n° 23/2020

The results were discussed based on logical analysis, and this option may present some difficulties, especially concerning the rationality of empirical beliefs [63]. Empirical beliefs can be false even if they have been rationally and logically created. If a logical error is possible, there is a factual dispute about which logic is correct [63]. Thus, to overcome any doubts, data were collected from as many documents/studies as possible, aiming to discuss the results presented.

The type of reasoning/form of research used was the inductive method, which allows inferring generalisations for the population based on certain data [64].

## 3 Results and discussion

Regarding our first research question, between 2008 and 2010, four PPP Hospital contracts were signed, covering the design, construction, financing, and operation of the new hospitals of Cascais, Braga, Vila Franca de Xira, and Loures, being the first three replacing existing units. In general terms, it is crucial to analyse the starting point of the four PPP projects in terms of expected savings, i.e., the possible *VfM* for the State. Overall, Table 3 summarises the savings estimated at the time the management contracts were signed, i.e., in the *ex-ante* moment [65].

**Table 3:** Estimated savings.

PPP Hospital	PSC (€)	BAFO (€)	Estimated Savings (€)	Estimated Savings (%)
**Vila Franca de Xira**	480,000,000	352,900,000	127,100,000	26.5
**Cascais**	312,299,000	269,825,000	42,474,000	13.6
**Loures**	643,430,000	443,329,000	200,101,000	31.1
**Braga**	987,627,000	641,504,000	346,123,000	35.0
**Total**	**2,423,356,000**	**1,707,558,000**	**715,798,000**	

The estimated savings were calculated considering the difference between the Best And Final Offer (BAFO), which translates the final proposal from the private partner that was adjudicated by the State, and the PSC, meaning the cost supported by the State if the public partner provisioned the service. The ex-ante moment represents the first indication of an expected positive *VfM* for the State.

In the *ex-post* analysis of different sources, several conclusions were found regarding the *VfM* evaluation for the various PPP Hospitals. Table 4 summarises all the findings produced by the State entities.

**Table 4:** Actual savings.

PPP Hospital	*VfM (€)*	Period	Source
**Vila Franca de Xira**	56,500,000	2011 to 2017	UTAP [66]
	30,000,000	2013 to 2017	AOC [56]
**Cascais**	40,400,000	2011 to 2015	UTAP [67]
	29,000,000	2011 to 2015	AOC [65]
**Loures**	167,100,000	2012 to 2017	UTAP [68]
	79,800,000	2013 to 2017	AOC [65]
**Braga**	199,100,000	2011 to 2015	UTAP [69]
	75,000,000	2012 to 2015	AOC [65]
**Total According to AOC**	**213,200,000**		
**Total According to UTAP**	**463,100,000**		

Thus, it is possible to ascertain that the State obtained overall savings of around 213 M€ according to the AOC and 463 M€ according to UTAP. The differences between the obtained *VfM* values (UTAP and AOC) results from the period analysed.

The literature [e.g., 23, [70]] indicates that the PPP model applied to the health sector is seen as a mechanism to achieve greater efficiency and lower costs during the life cycle of the projects. Still, the empirical evidence to date does not support the theoretical expectation of such savings. However, even though the data analysed only allows a partial estimate of the results, there seems to be an effective savings trend for the State, contradicting this evidence.

On the other hand, our results align with what was advocated by Tang et al. [71]. These authors argued that one of the main advantages of applying partnership models for the public partner is cost-effectiveness (obtaining *VfM*) and better service delivery. In contrast, for the private partner, one of the main advantages will be better investment potential and a reasonable profit [10,72].

Considering that all the reports analysed do not calculate savings for the whole period of implementation of the management contracts, we can extrapolate that the global savings for the entire period of the management contracts will tend to be higher than those forecast at the time of the initial estimates. If it is estimated average savings are equal to the average obtained in each PPP hospital, it can be expected higher global savings for the State. This idea is also defended by the AOC [65] when it states that “considering the total period of execution of the contracts will tend to produce higher values for the estimated savings of the State” (p.22).

Torchia et al. [31] argued that a PPP project is successful when it results in a positive *VfM* for the taxpayer, an idea also defended by Marques and Berg [73]. This study shows partial results toward the Portuguese State obtaining a positive *VfM* for the taxpayer, as all the projects in the sample proved to have an investment value lower than the PSC measured in an ex-ante phase.

One can verify an *ex-ante* assessment of potential savings for the Portuguese State, which was also proven *ex-post*, contradicting the idea defended by Silva [74] and António [75]. They indicated that using the *VfM* and PSC methodologies is ambiguous and prone to biased manipulations, generating, *a posteriori*, an even more significant burden for the public purse. Let's analyse the justifications pointed out by António [75] to consider the *VfM* and PSC methodologies biased. One can verify that they are not corroborated by the present research, namely when the author states: “Reality reveals to us, by the poor results pointed out by the AOC, that these preliminary analyses of the economic-financial content of feasibility and sustainability of the PPP in health, were not properly carried out (p.14)". This research shows precisely the opposite, with the same AOC [65] stating that choosing the PPP model was supported by a perspective of obtaining *VfM* for the State, which was estimated by comparing the expected charges with the PPP and the PSC. Additionally, regarding the use of the PSC, UTAP [66] clearly states in its report that:

It becomes possible to conclude that the charges borne by the public partner, in the period between June 2011 and December 2017, appear to be lower than the costs that the Portuguese State would bear with the clinical management activity of the Vila Franca de Xira Hospital, if it had opted for the public management model of that activity, taking into account the cost structure provided for in the initial PSC, duly adjusted by the actual values of the operational activity in that period (p.122).

Thus, UTAP concluded that the model performed well, but it also referred to the correct use of the PSC for the calculation of the respective *VfM*. As an answer to the first research question, one can conclude that the results were positive, undoubtedly generating a *VfM* for the State and, therefore, for the taxpayer.

It is important to point out the conclusion of AOC [65], supported by the data produced by UTAP [[66], [67], [68], [69]], which refers to the positive assessment of the 4 PPP hospitals, concluding that there is a *VfM* for the Portuguese State, having recommended that the Portuguese State adopt PPP models, instead of an internalization scenario, as a continuity solution for the clinical management of the 4 PPP hospitals. Additionally, to the positive *VfM*, Rodrigues [76] also concluded that all 4 PPP hospitals reached the production levels and complied with the contractual stipulations.

Regarding the second research question, the requirement of performance indicators presented in the Management contracts of PPP Hospitals differs from the quality indicators required and measured in the PMH, which translates into an increase in quality for users served by the PPP model. This idea was reinforced when the AOC [65] concluded: “More demanding quality standards protect users of hospitals managed in PPP than those applied in the monitoring of PMH” (p.7). Additionally, the managing entities of PPP hospitals must implement quality management systems that include aspects such as the annual accreditation of hospitals by certified international entities.

We cannot fail to mention the conclusions of Nunes [45], who found that PPP hospitals were more efficient between 2011 and 2016 than PMH. The same author also concluded that PPP hospitals, for the same period, showed better productivity results than PMH. These findings reinforce the idea, referred to in the literature, that private partner management practices can provide services at a lower cost [e.g., [77]] and in an economically efficient way [e.g., [[78], [79]]].

According to Grimsey and Lewis [19], the PPP model offers a solution to the problems of the public procurement model. Still, it is not yet possible to determine whether PPP projects perform better than projects developed under a public management model (PMH). However, our results effectively allow us to compare the performance of the PPP model with the PHM model. Table 5 summarises all the indicators analysed, presenting a performance average during ten years of operation. One can see a superior performance of the PPP model in most of the indicators analysed.

**Table 5:** Summary results - performance indicators.

			Group B		Group C		Group C		Group D	
Dimension	Indicator	Best performance for each Hospital Group	PPP VFX	PMH Figueira	PPP Cascais	PMH Setúbal	PPP Loures	PMH Leiria	PPP Braga	PMH Évora
Health Care Performance (%)	A	Highest value of percentage of surgeries performed in outpatient clinics for outpatient procedures.	83.42	78.27	86.84	80.80	83.23	83.67	84.56	75.66
	B	Lowest percentage of readmissions within 30 Days.	7.54	9.18	5.88	8.26	7.58	8.02	6.80	5.75
	C	Lowest percentage of hospitalisations with a delay of more than 30 days.	2.58	2.85	2.12	3.36	3.47	2.10	3.15	3.00
	D	Smallest percentage of births by caesarean section	29.16	n.a	27.68	30.19	22.12	28.92	29.08	34.44
	E	Highest percentage of hip fractures with surgery performed within the first 48 Hours	48.39	49.90	82.07	61.55	39.80	36.79	41.37	19.74
	F	Smallest percentage of births by caesarean section in single, cephalic, full-term pregnancies.	29.10	n.a	26.29	29.45	20.31	30.54	27.44	32.27
	G	Smallest percentage of first caesareans in single, cephalic, full-term pregnancies.	73.68	n.a	72.57	73.11	65.75	76.44	74.44	72.94
	H	Highest percentage of vaginal deliveries after caesarean in single, cephalic, full-term pregnancies.	9.26	n.a	8.95	1.29	4.08	6.29	9.95	5.77
Costs Performance (€)	I	Lowest operating cost per standard patient	1 845,51	2 776,18	n.a	2 585,38	2 200,00	2 429,06	1 682,71	2 459,46
Access Performance (%)	J	Highest percentage of consultations carried out in time.	73.85	89.23	65.63	65.44	56.05	72.73	64.13	65.40
	K	Highest percentage of enrolees with waiting times less than the GMRT	78.72	97.02	90.99	73.81	80.03	87.35	72.36	75.80

Regarding group B, where PPP VFX is compared with Figueira PMH, only three out of 11 indicators show a better performance of the PMH unit, namely *"% Hip fractures with surgery performed within the first 48 h*”, "*% 1*st *consultations carried out in time*”, and *"% Surgical enrolment list, within the Guaranteed Maximum Response Times (GMRT)*". This higher performance registered for the three indicators might be explained because PMH Figueira is located outside the Lisbon metropolitan area, thus leading to a lower patient incoming.

In Group C, Cascais PPP has shown better performance on all the 11 indicators analysed compared with Setúbal PMH. In the other comparison, Loures PPP outcomes Leiria PMH in six of the 11 indicators, being the case with less significant performance results between the PPP and PMH models. Once again, the Leiria PMH is geographically located outside the Lisbon metropolitan area, while Loures PPP operates in the centre of the capital region. This fact might influence the Hospital performance results.

Finally, the comparison between Braga PPP and Évora PMH regarding group D. In this case, the PMH unit only slight outperforms the PPP unit on four of the 11 indicators, namely "*% of Readmissions within* 30 Days”, "*% of First Caesarean sections in single, cephalic, term pregnancies*”, "*% 1*st *Consultations Carried out in Time*” and "*% of Surgical Enrolment List, within the GMRT*”. Once again, the unit's location might influence the performance in favour of the PMH unit, which is located in an area of high population density, unlike Évora PMH in the south of Portugal.

Concerning the quality of service provided by the four PPP hospitals, this study concluded that in most indicators analysed, the performance of these hospital units was superior to the performance registered in the four comparable PMH of the same cluster. This conclusion is in line with the findings of the work developed by Ferreira and Marques [80]. They used robust benchmarking methodologies and recent data on Portuguese hospitals (between 2012 and 2017) to demonstrate that, in reality, PPP hospitals can provide health services with performance levels that are at least as good as those of PMH. Additionally, it is important to link this conclusion to the results reported by Carvalho & Rodrigues [81] where the PPP hospital patients were more satisfied than those from PMH. This study's results are also in line with the AOC [65] findings, which showed that PPP hospitals were generally more efficient than the comparable PMH. Notwithstanding these findings, it is crucial to take into account that PPP hospitals are subject to an annual negotiation with the Contracting Public Entity (CPE) regarding the Hospital's output, regardless of the installed capacity [53], and this fact negatively influences the performance of the analysed indicators. Thus, answering the second research question, one may conclude that, in fact, and in general, PPP hospitals presented a superior performance, notwithstanding the limitations imposed by the annual negotiation with the CPE.

## 4 Conclusion

One concludes that, in Portugal, the PPP model, applied to the health sector, appears to be advantageous regarding the *VfM* results and the quality-of-service provision. This study clearly shows that the PPP model provides a lower cost ex-ante and a *VfM* that saved around 465 M€ for the public partner in the four PPP hospitals.

Concerning the quality of service provided by the four PPP hospitals, one concludes that the PPP model performance was superior to the performance registered in the four comparable PMH of the same cluster in most of the indicators analysed. In other words, there was no worse quality of service provided by the PPP model; quite the contrary.

The PPP model must go through its entire life cycle and achieve its economic, financial, and social objectives to be considered successful. These objectives are materialised in providing public goods in sufficient quantity and quality, improving efficiency and the *VfM*, and increasing equity in the less developed areas such as health, stakeholders' satisfaction, and conservation of resources. However, notwithstanding the *VfM* generated by the PPP model in the four hospitals analysed in Portugal and lower construction costs and superior performance compared to PMH, the Portuguese government continues to revert the PPP management model to the model managed only by the State. They seem not to use performance evaluation measures but only their ideological principles. There is still the decision regarding the Cascais PPP that is waiting to be unveiled.

One of the limitations of the present research relates to the absence of data regarding some indicators that were part of the selected dimensions. As referred by the General Inspection of Health Activities [82], the CAHS does not have complete information regarding the cost performance indicators for three PPP hospitals (Cascais, Loures, and VFX). Lastly, the limitation related to the lack of studies carried out on hospital PPPs in Portugal must be emphasised. This fact has prevented us from having a more extensive discussion.

Considering that data identify the PMH's performance by hospital cluster, it is suggested that the public and private institutions do further investigation in all hospitals to understand whether the application of the PPP model could contribute to improving those performances. Considering that three PPP hospitals (VHX, Loures, and Cascais) came to an end in 2022, it will be relevant to analyse their evolution in future studies in terms of economic, financial, and social results in comparison to the last ten years of management under the PPP model. This study questions the decision to end the PPP model in Portuguese hospitals because it may not be the best for the State and the population. Moreover, ideological conviction is not always the best for the country, and political decisions must also be based on serious and independent studies.

The research results might be used by all decision-makers and practitioners that are in the process of deciding or analyzing which model to adopt to address the infrastructure gap, especially in terms of constructing and operating new hospitals. It is possible to assess that a crucial implication of the present research is linked to the PPP hospitals’ better performance allied with *VfM* to the state, which can shade a new light on the theoretical rhetoric presented in the literature.

## Author contribution statement

Nuno Rodrigues: Conceived and designed the experiments; Performed the experiments; Analysed and interpreted the data; Contributed reagents, materials, analysis tools or data; Wrote the paper. João M.S. Carvalho: Conceived and designed the experiments; Analysed and interpreted the data; Contributed reagents, materials, analysis tools or data; Wrote the paper.

## Funding

This work is financed by national funds through 10.13039/501100001871FCT - Foundation for Science and Technology, I.P., within the scope of the project « UIDB/05105/2020» of REMIT – Research on Economics, Management and Information Technologies.

## Additional information

No additional information is available for this paper.

## Declaration of competing interest

The authors declare that they have no known competing financial interests or personal relationships that could have appeared to influence the work reported in this paper.
